# Photon-counting CT: technical features and clinical impact on abdominal imaging

**DOI:** 10.1007/s00261-024-04414-5

**Published:** 2024-06-18

**Authors:** Hiromitsu Onishi, Takahiro Tsuboyama, Atsushi Nakamoto, Takashi Ota, Hideyuki Fukui, Mitsuaki Tatsumi, Toru Honda, Kengo Kiso, Shohei Matsumoto, Koki Kaketaka, Yukihiro Enchi, Shuichi Kawabata, Shinya Nakasone, Noriyuki Tomiyama

**Affiliations:** 1 Department of Radiology, Osaka University Graduate School of Medicine, Suita, Japan; 2 Department of Medical Physics and Engineering, Osaka University Graduate School of Medicine, Suita, Japan; 3 Division of Radiology, Department of Medical Technology, Osaka University Hospital, Suita, Japan

**Keywords:** Photon-counting CT, Abdomen, Liver, Pancreas, Metabolic dysfunction-associated steatohepatitis (MASH), Iron overload

## Abstract

Photon-counting CT has a completely different detector mechanism than conventional energy-integrating CT. In the photon-counting detector, X-rays are directly converted into electrons and received as electrical signals. Photon-counting CT provides virtual monochromatic images with a high contrast-to-noise ratio for abdominal CT imaging and may improve the ability to visualize small or low-contrast lesions. In addition, photon-counting CT may offer the possibility of reducing radiation dose. This review provides an overview of the actual clinical operation of photon-counting CT and its diagnostic utility in abdominal imaging. We also describe the clinical implications of photon-counting CT including imaging of hepatocellular carcinoma, liver metastases, hepatic steatosis, pancreatic cancer, intraductal mucinous neoplasm of the pancreas, and thrombus.

**Graphical Abstract:**

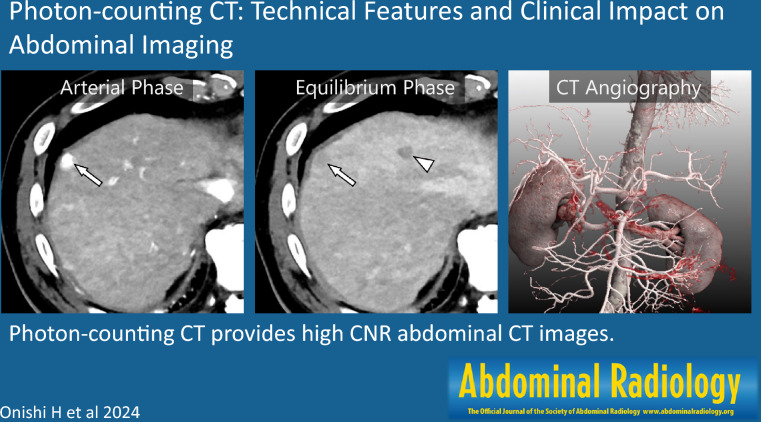

## Introduction

In abdominal imaging, CT has played a very important role in various diseases and conditions. In the field of oncology, CT has been used for detection, staging and preoperative planning of various tumors [1, 2]. In chronic diseases, CT has also been used to evaluate substances present in the organs, such as fatty deposition in the liver in metabolic dysfunction-associated steatohepatitis or hepatic iron deposition in iron overload condition [3].

Since the introduction of clinical CT in the early 1970s, CT have has advanced rapidly [4, 5]. One of the milestones in the history of CT was the advent of multidetector CT, which allowed for improved spatial resolution and wider image coverage, both of which contributed greatly to tumor imaging, including hepatocellular carcinoma and pancreatic neoplasia in many aspects [6, 7]. In addition, the advent of dual-energy CT made it possible to accurately quantify substances in organs, such as lipids [8]. On the other hand, virtual monochromatic imaging with dual-energy CT has improved image contrast, which has also contributed to improved tumor detection [9].

More recently, photon-counting detector technology has been introduced into clinical CT [10]. Designed to overcome the contrast and spatial resolution limitations of conventional CT and to reduce radiation dose, this technology represents one of the most significant innovations in CT equipment to date. The photon-counting detector can measure the number of photons and the energy of each photon, allowing the acquisition of images with high spatial resolution and virtual monochromatic contrast [11]. Thus, photon-counting CT has tremendous potential, but it has only been clinically available for a short time, and studies of its diagnostic ability and clinical utility for abdominal lesions are limited at this time, and further research and experience are needed before its benefits can be understood.

We offer here a review of the actual clinical operation of photon-counting CT and its diagnostic utility in abdominal imaging.

## Technical overview of photon-counting detector

Photon-counting CT has a detector with a completely different mechanism than conventional CT. Conventional CT detectors convert X-rays into light, and then this light is converted into electrical signals using a photodiode, but photon-counting detectors can convert X-rays directly into electrons (charge cloud) and receive them as electrical signals [11].

Photon-counting CT has two major advantages over conventional CT equipped with energy-integrating detectors. One is better dose efficiency, and the other is better spatial resolution.

Photon-counting CT does not require a detector septum, thus increasing the aperture area [11]. There is no electrical noise because subthreshold electrical signals are eliminated in photon-counting CT [12]. In addition, there is no "energy weighting effect" in photon-counting CT, *i.e.* the scintillation light in an energy-integrating detector becomes stronger and the output increases in proportion to the energy of the X-ray photon, but in photon-counting detector the output is constant regardless of the energy of the X-ray photon [13]. As a result, sensitivity is relatively high in the low energy range, resulting in improved soft tissue contrast in photon-counting CT [14]. These characteristics of the photon-counting detector contribute to improved dose efficiency [15].

Each detector pixel of the photon-counting detector is smaller than that of conventional detectors [12]. In addition, the higher resolution results in increased image noise, but this effect can be reduced by improving dose efficiency. For these reasons, photon-counting CT can improve the spatial resolution of CT images [16].

## Radiation dose reduction opportunities

**Fig. 1:**
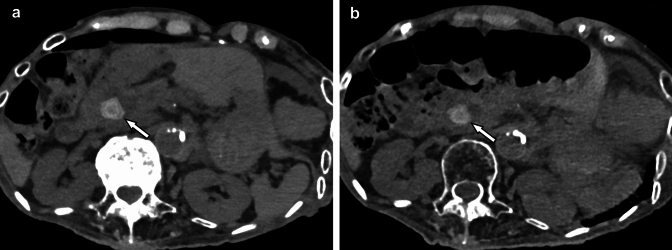
Non-contrast abdominal CT images of a patient with choledocholithiasis scanned with photon-counting CT (**A**) and conventional dual-source CT (single-energy mode) (**B**) on different days. Image noise was apparently lower with photon-counting CT than with conventional CT, even though the radiation dose was lower with photon-counting CT (CTDI_vol_: 7.5 mGy) than with conventional CT (11.9 mGy). The layered calcified stone in the common bile duct (arrows) is visualized more precisely with photon-counting CT

**Fig. 2:**
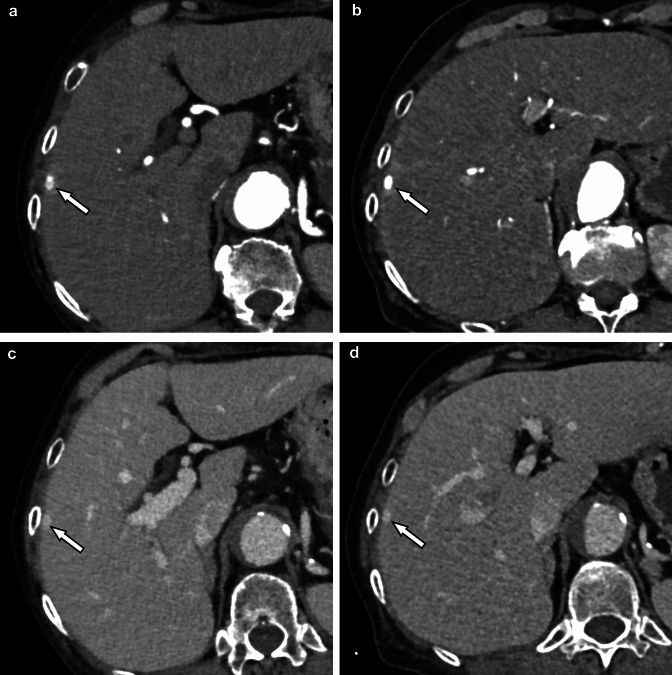
Contrast-enhanced dynamic CT images during the arterial dominant phase (**A, B**) and the equilibrium phase (**C, D**) of the liver scanned with photon-counting CT (**A, C**) and conventional dual-source CT (single-energy mode) (**B, D**) on different days. A small hemangioma is seen under the capsule of the right lobe of the liver (arrows). Although the radiation dose of the photon-counting CT (CTDI_vol_: 4.6 mGy) was coincidently half of that of the conventional CT (9.2 mGy), the image quality remained comparable

Because photon-counting detectors have no detector septum, no electrical noise, and no “energy weighting effect”, they are inherently more dose efficient. Therefore, photon-counting CT can achieve the same spatial resolution and image noise as conventional CT at a lower radiation dose (Fig. 1, 2). Photon-counting CT provides a significant dose reduction (approximately 32%) in contrast-enhanced abdominal CT while maintaining image quality similar to that of second-generation dual-source CT [17]. Decker et al. reported that image noise was significantly lower, and iodine signal-to-noise ratio was significantly higher, and subjective image quality was higher and conspicuity was better for the renal pelvis, ureters, and mesenteric vessels with low-dose photon-counting CT (mean CTDI_vol_ 1.61 mGy) compared with low-dose second-generation dual-source CT (mean CTDI_vol_ 1.45 mGy) [18].

Photon-counting CT is also advantageous for imaging patients with large body habitus. When dual-energy imaging is performed on obese patients with conventional detector CT, the overall image quality is reduced due to the lack of photons at low kVp acquisition [19]. Hagen et al. reported that contrast-enhanced abdominal CT using photon-counting CT in obese patients achieved similar or improved image quality and significant dose reduction compared with conventional single-energy CT [20].

## Iodine contrast dose reduction opportunities

Improved contrast-to-noise ratio in photon-counting CT may provide an opportunity to reduce the contrast dose required for abdominal imaging. In the phantom study, a 30–37% improvement in the iodine contrast-to-noise ratio was observed, suggesting that there is room for a reduction in the contrast dose used [21]. Hagen et al. reported that photon-counting CT (non-spectral images at 120 kVp) allows a significant 27% contrast dose reduction with a comparable contrast-to-noise ratio, in addition to a radiation dose reduction of up to 34% compared to conventional CT at 120 kVp (single energy mode) in obese patients [22].

## Scan modes

The currently available clinical photon-counting CT has three main scan modes, including ultra-high resolution (UHR) mode, multi-energy mode, and dual-source mode. The UHR mode provides the highest spatial resolution images. The UHR mode uses 120 rows with a slice thickness of 0.2 mm, resulting in a total width of 24 mm. Multi-energy mode is the imaging method capable of producing virtual monochromatic images at standard scan speeds. The use of dual-source mode allows for a very fast scan speed. Dual-source mode uses two detectors, each with 144 rows and a slice thickness of 0.4 mm. This results in a total width of 115.2 mm. Each imaging mode should be used according to the purpose of the examination. Of these, the multi-energy mode is frequently used for abdominal scanning because of the many potential applications of spectral imaging with this mode, including the ability to generate different levels of monoenergetic images [22, 23]. Meanwhile, the use of the dual-source mode, which allows for very fast scanning, can be considered for patients who have difficulty holding their breath. High-speed scans can reduce motion artifacts associated with respiratory motion.

## Image reconstruction

**Fig. 3:**
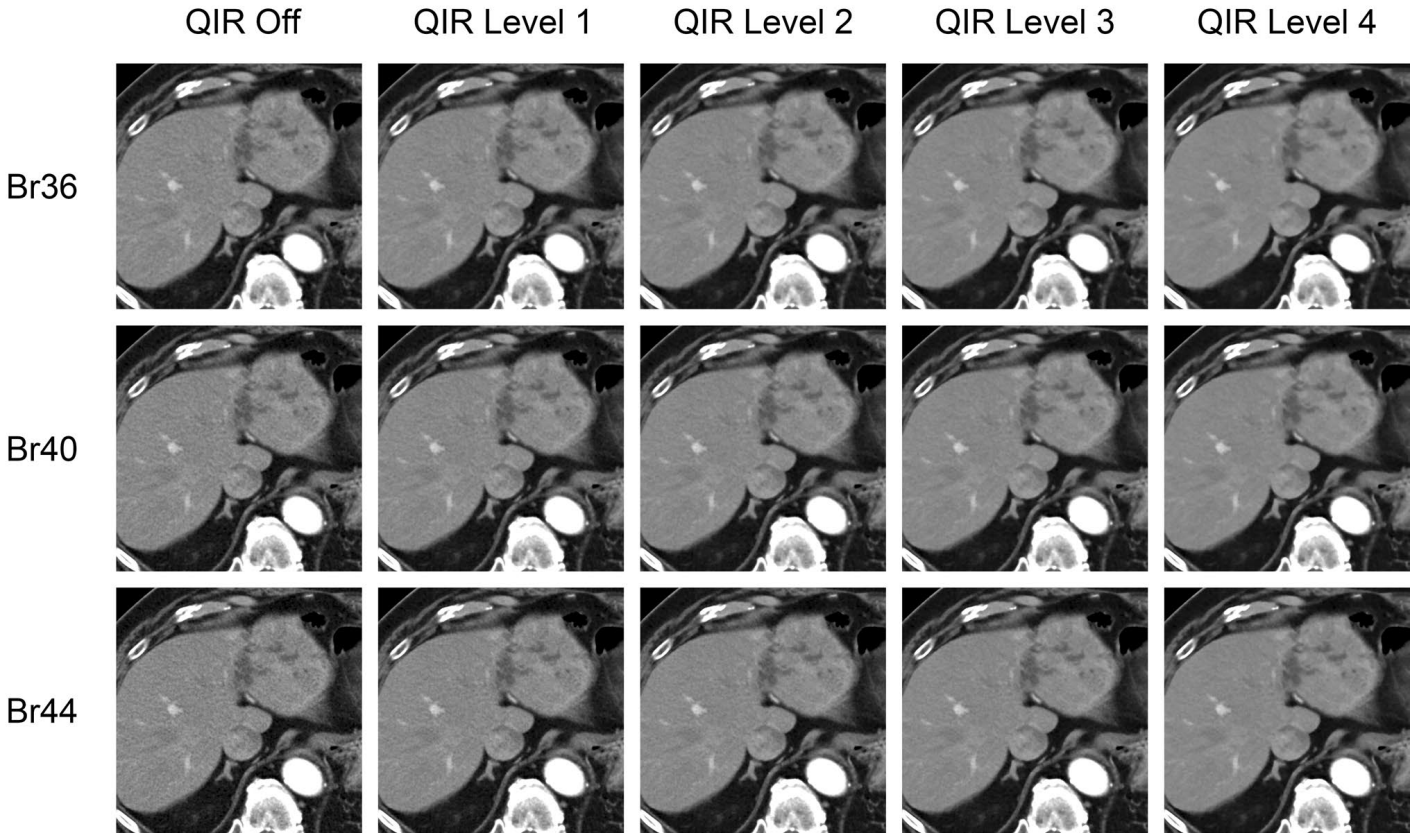
Photon-counting CT images with different reconstruction settings are shown aligned. The higher the resolution index (Br36, Br40, Br44), the higher the resolution, but the higher the image noise. The higher the level of the Quantum Iterative Reconstruction (QIR) algorithm, the lower the image noise

The photon-counting CT scanner is equipped with a wide variety of reconstruction kernels (Table 1). In general, the body regular (Br) kernel is used for abdominal CT images [24]. The numbers in the kernel indicate the index numbers. The larger the resolution index number, the higher the resolution, but the image noise increases. Therefore, it is necessary to select an appropriate value, while taking into account the degradation of image quality due to increased image noise. However, image noise may also be affected by the Quantum Iterative Reconstruction (QIR) algorithm condition settings described below, so both the kernel and QIR algorithm settings need to be considered together (Fig. 3). For CT angiography, the Body Vascular (Bv) kernel is recommended because it reduces blooming artifacts caused by objects with very high attenuation. Spectral analysis requires images reconstructed using the Quantitative Regular (Qr) kernel. For the evaluation of hepatocellular carcinoma in photon-counting CT, softer reconstruction kernels are superior in terms of noise and image quality, but no significant differences in image contrast and lesion conspicuity are found between the different reconstruction kernels [25].

**Table 1:** The list of image reconstruction kernels at photon-counting CT

		Kernel Family & Kernel Group						
		Body (B)			Head (H)			Quantitative (Q)
		l (lung)	r (regular)	v (vascular)	c (crisp)	r (regular)	v (vascular)	r (regular)
Resolution	36		Br36	Bv36		Hr36	Hv36	Qr36
Index	40		Br40	Bv40	Hc40	Hr40	Hv40	Qr40
	44		Br44	Bv44	Hc44	Hr44	Hv44	Qr44
	48		Br48	Bv48		Hr48	Hv48	Qr48
	56	Bl56	Br56	Bv56		Hr56	Hv56	Qr56
	60	Bl60	Br60	Bv60		Hr60	Hv60	Qr60
	64	Bl64	Br64	Bv64		Hr64	Hv64	Qr64
	68		Br68	Bv68		Hr68	Hv68	Qr68
	72		Br72	Bv72		Hr72	Hv72	Qr72
	76		Br76	Bv76		Hr76	Hv76	Qr76
	80		Br80	Bv80		Hr80	Hv80	Qr80
	84		Br84	Bv84		Hr84	Hv84	Qr84
	89		Br89	Bv89		Hr89	Hv89	Qr89
	92		Br92			Hr92		
	96		Br96			Hr96		
	98		Br98			Hr98		

Note: Kernels with a resolution index of 80 or higher can only be used in UHR mode

Although photon-counting CT has excellent dose efficiency, a denoising algorithm may be essential for abdominal CT imaging. A high-intensity iterative reconstruction algorithm improves image quality by reducing image noise in abdominal photon-counting CT [23]. There are four levels of QIR strength, where level 1 is the weakest and level 4 is the strongest [23]. A high strength level corresponds to level 3 or 4. Especially in images with thin slice thickness, image noise is significant, so the iterative reconstruction algorithm at the highest strength level is very effective in reducing image noise. The currently available clinical photon-counting CT scanner is equipped with an iterative reconstruction algorithm (QIR), but deep learning image reconstruction techniques are not yet installed.

## Virtual monochromatic imaging

**Fig. 4:**
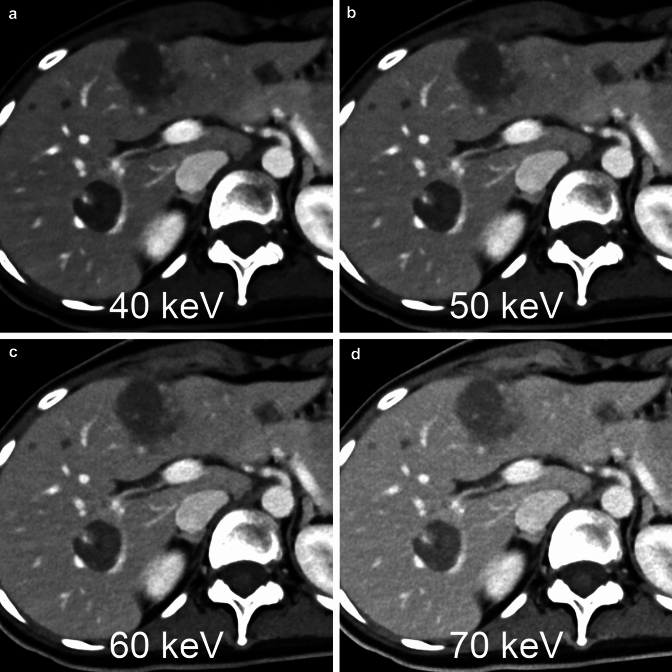
Using a photon counting detector, virtual monochromatic images of any energy level from 40 to 190 keV can be generated from 120kVp single-energy raw data. The lower the keV setting of the virtual monochromatic image, the higher the contrast between the liver lesion and the liver parenchyma

One of the major advantages of photon-counting CT in abdominal examinations is that it provides virtual monochromatic images with high image quality. The photon-counting detector is able to determine the energy intensity of the photons, making it possible to generate virtual monochromatic images from single-energy acquisition data at, for example, 120 kVp (Fig. 4). The minimum and maximum energy level settings for virtual monochromatic images in this CT system are 40 and 190 keV, respectively [24, 26]. The low-keV virtual monochromatic images with the photon-counting CT can provide superior performance over conventional single-energy (non-spectral) CT in arterial phase imaging, including significantly higher contrast-to-noise ratio, better image quality, and lesion conspicuity [27]. The low-keV virtual monochromatic images with the photon-counting CT have a significantly higher contrast-to-noise ratio with similar subjective image quality as compared to conventional single-energy dual-source CT at identical radiation dose [24].

The mainstream of conventional dual-energy CT is to scan with two different energy X-ray beams, for example 80 kVp and 140 kVp, and analyze the data to generate virtual monochromatic images [28]. Therefore, prior to scanning, a decision had to be made whether to perform dual-energy imaging or normal single-energy imaging. The design of the photon-counting detector allows virtual monochromatic images to be reconstructed from single-energy data acquired at 120 kVp or 140 kVp. There is no need to decide on the type of acquisition before scanning, considering whether the virtual monochromatic imaging is needed or not [19]. For the virtual monochromatic images at energies below 60 keV, the contrast-to-noise ratio of the photon-counting CT images was generally higher than that of the corresponding energy-integrating detectors dual-energy CT images [29].

## High spatial resolution

Photon-counting CT can improve spatial resolution due to the nature of its detector structure. When scanning in UHR mode, images with a thickness of 0.2 mm can be obtained. The image matrix size can also be output at 1024 × 1024, and when the field-of-view is reduced to 20 cm, it is also possible to generate 0.2 mm isotropic voxel image data. For high-contrast structures such as the lungs and temporal bones, the extremely high spatial resolution allows fine structures to be visualized in detail. In abdominal CT, the high spatial resolution characteristic of photon-counting CT can be exploited in CT angiography generated from early arterial phase images. Additional applications, such as the detection and staging of pancreatic cancer and cystic pancreatic lesions (*i.e.*, intraductal papillary mucinous neoplasms), could potentially benefit from this new capability [19]. Photon-counting CT also has the potential to improve the detection and determination of the composition of small urolithiasis [30]. This diagnosis might be important for patients with renal stones, as detection of these small stones could allow for early clinical intervention [30].

## Scanning protocols optimized for the abdominal diseases

Virtual monochromatic images are available with acquisition at 120 kVp or 140 kVp of X-ray tube voltage. Because acquisition at 140 kVp provides the best possible spectral separation at the detector level [31], Schwartz et al. acquire all adult abdominal CT images at 140 kVp [19]. When performing abdominal photon-counting CT, many radiologists are concerned with determining which keV setting of virtual monochromatic image reconstruction is optimal for diagnosis. The 40 keV images have the best contrast-to-noise ratio for abdominal organs, vascular structures, and hypovascular liver metastases [24, 26]. However, CT image quality may be affected by factors other than the contrast-to-noise ratio. Racine et al. reported that the highest detectability was found at 65 and 70 keV for both hypoattenuating and hyperattenuating objects, regardless of phantom size and radiation dose, in the phantom study, which was performed assuming the detection of hepatocellular carcinoma [32]. Higashigaito et al. reported that the overall image quality of abdominal CT tended to be higher at 50 keV and 60 keV compared with energy-integrating detector CT, whereas the overall image quality was lower at 40 keV compared with energy-integrating detector CT in the clinical study [24]. Further studies may be needed to determine the optimal keV setting for diagnostic abdominal CT using photon-counting CT in clinical practice.

Abdominopelvic CT is usually performed in a single breath hold. Acquisition of abdominopelvic CT with the thinnest collimation (0.2 mm × 120 rows, UHR mode) is more difficult due to longer scan times, so it is generally performed with routine collimation (0.4 mm × 144 rows) [33]. However, when acquired with this setting, the minimum slice thickness of the images is 0.4 mm.

An example of the acquisition and reconstruction parameters for a scan of the abdomen using photon counting CT is shown in Table 2.

**Table 2:** Example of acquisition and reconstruction parameters for abdominal scan using photon-counting CT

Parameters	
Voltage (kVp)	120 or 140
Collimation (mm)	144 × 0.4 or 120 × 0.2
Pitch	0.8
Rotation time (s)	0.5
Kernel	Br40 or Br44*
QIR level	2 or 3
Energy level (keV)	70 (and 50)
Matrix	512 × 512

Note: * For spectral post processing, the images reconstructed with the kernels Qr (quantum regular) are needed

## Clinical implications for the diagnosis of abdominal diseases

Virtual monochromatic imaging technique on a photon-counting CT scanner achieves higher image contrast and lower image noise, allowing precise delineation of lesions and has the potential to improve detection of liver and pancreatic tumors and peritoneal lesions. As mentioned above, virtual monochromatic images with photon-counting CT are expected to improve CNR compared to virtual monochromatic images with conventional CT [29]. In the phantom study, photon-counting CT significantly improved the detection of focal liver lesions smaller than 1 cm, especially at low radiation dose, compared to conventional dual-energy CT [32]. The high spatial resolution and high contrast images with photon-counting CT are also effective for CT angiography to delineate small visceral arteries.

Virtual noncontrast imaging based on the spectral data from a photon-counting detector is also a valuable technique in the abdominal CT. Virtual noncontrast images with photon-counting CT have similar CT attenuation accuracy to true noncontrast images [34]. Importantly, the photon-counting CT significantly outperformed conventional dual-energy CT in terms of quantification accuracy, even after adjusting for confounding variables such as radiation dose and patient size [34]. In addition, Mergen et al. demonstrated that the CT attenuation difference was within 10 H.U. in 95% of the measurements [35].

### Liver imaging

#### Hepatocellular carcinoma

**Fig. 5:**
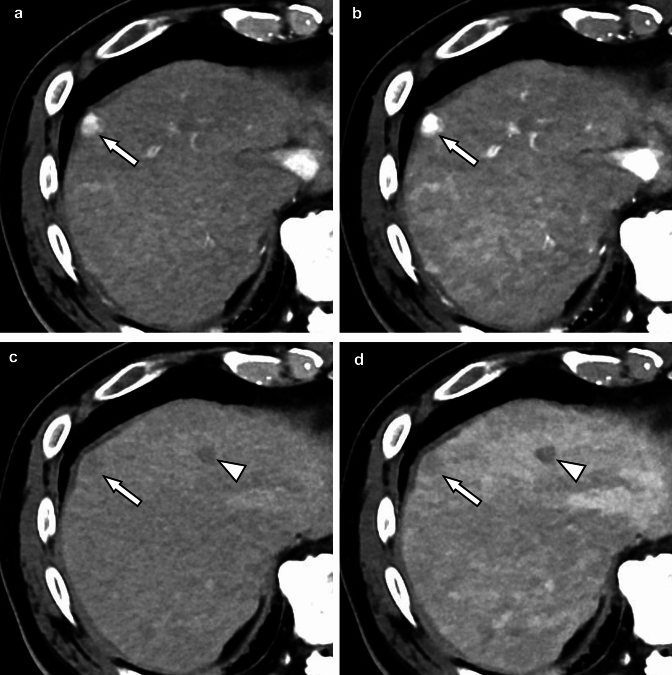
Contrast-enhanced photon-counting CT images during arterial dominant phase (**A, B**) and equilibrium phase (**C, D**) at 70 (**A, C**) and 50 keV (**B, D**). Arterial enhancement and washout pattern of HCC are better visualized on 50 keV images (**B, D**) than on 70 keV images (**A, C**) in the arterial and equilibrium phases, respectively (arrows). The hypovascular nodule (arrowheads: suspected dysplastic nodule) is also more clearly delineated on the 50 keV image (**B**) than on the 70 keV image (**D**)

Hepatocellular carcinoma is the most common primary liver cancer [36] and one of the most malignant cancers with high morbidity and mortality [37]. In the diagnostic imaging of hepatocellular carcinoma, detailed evaluation of contrast enhancement on dynamic contrast-enhanced CT, such as early enhancement during the arterial phase and washout pattern during the equilibrium phase, provides very important clues [38]. The high contrast-to-noise ratio of the photon-counting CT makes it possible to clearly visualize subtle differences in contrast, which might contribute to improved diagnostic performance when evaluated in the future (Fig. 5).

#### Liver metastases

**Fig. 6:**
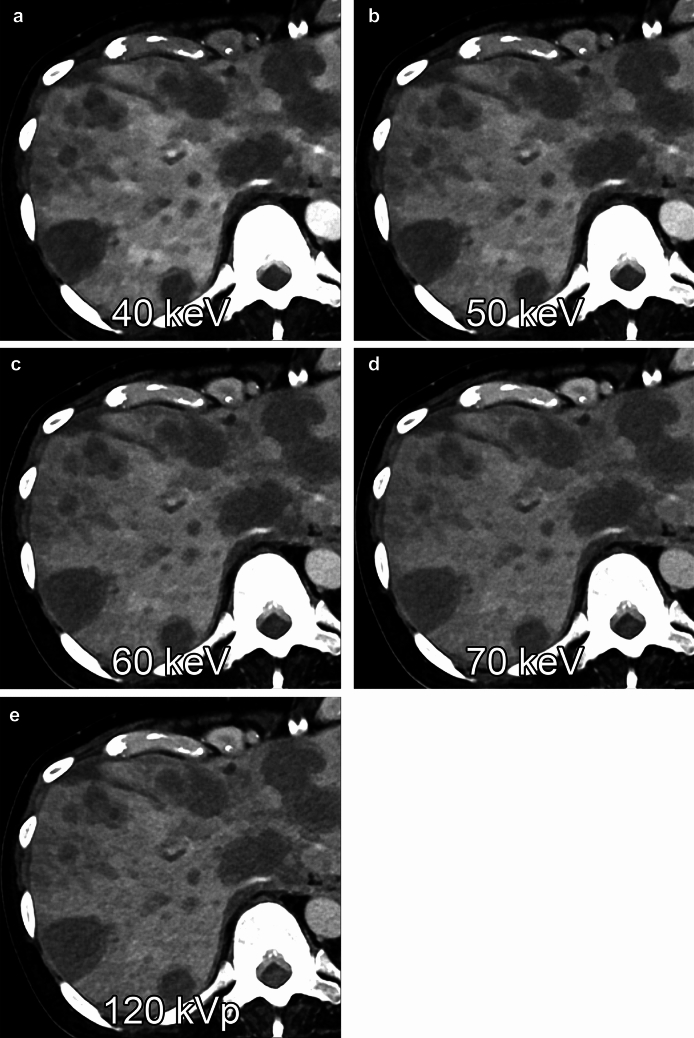
Multiple metastatic liver tumors from pancreatic cancer were shown on virtual monochromatic images at 40 (A), 50 (B), 60 (C), and 70 keV (D) and polychromatic image (*i.e.*, T3D) acquired with photon-counting CT at 120 kVp (E) during the portal venous dominant phase. The contrast-to-noise ratios between the liver metastasis and the liver parenchyma are 14.6, 11.9, 9.8, 8.5, and 7.4, respectively

Liver metastasis is the most common type of liver cancer, and most cases have hypovascular characteristics. Virtual monochromatic images with photon-counting CT show reduced image noise compared to conventional single-energy CT and improved conspicuity of hypovascular liver metastases at lower keV levels, and patients with a high body mass index benefit especially from photon-counting CT compared to conventional CT [26] (Fig. 6).

#### Hepatic steatosis

**Fig. 7:**
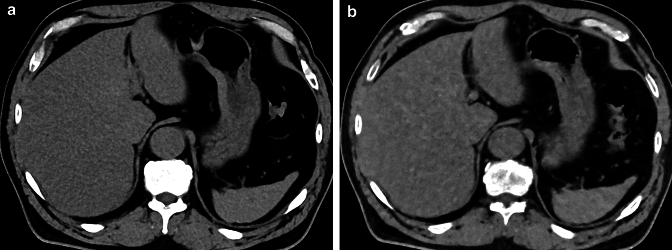
A true noncontrast CT image (**A**) and a virtual noncontrast CT image obtained from contrast-enhanced image data (**B**) acquired with photon-counting CT. Virtual noncontrast images with photon-counting CT have similar CT attenuation accuracy to true noncontrast images. In both images, the liver parenchyma is less attenuated than the spleen, indicating hepatic steatosis

Hepatic steatosis, including metabolic dysfunction-associated fatty liver disease and metabolic dysfunction-associated steatohepatitis, is a serious pathological condition of the liver that can progress to cirrhosis and hepatocellular carcinoma [39]. Estimation of intrahepatic fat is essential for the diagnosis of hepatic steatosis [40]. Photon-counting CT has been shown to be accurate for index-based liver fat estimation from virtual non-contrast images [41] and has the potential to increase the likelihood of incidental detection of liver steatosis on contrast-enhanced abdominal CT scans (Fig. 7). In addition, photon-counting CT can quantify intrahepatic fat even in the presence of iron overload [42].

### Pancreas imaging

#### Pancreatic *cancer*

**Fig. 8:**
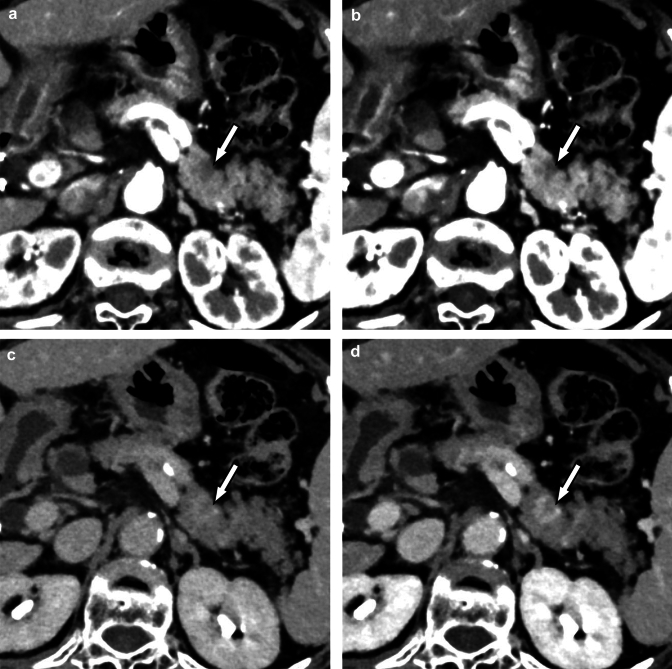
Pancreatic cancer is shown as a small hypovascular nodule in the tail of the pancreas (arrows) on the pancreatic parenchymal phase (arterial phase) photon-counting CT images at 70 (**A**) and 50 keV (**B**). Meanwhile, enhancement of the tumor (arrows) is shown during the equilibrium phase at 70 (**C**) and 50 keV (**D**). The delayed enhancement is more evident in the 50 keV image (D) than in the 70 keV image (C). Small pancreatic cancers are often not visualized in the pancreatic parenchymal phase but are visualized as delayed-enhancement lesions only in the equilibrium phase. Low-keV images obtained with photon-counting CT may help in the early detection of pancreatic cancer

Pancreatic cancer has a significantly poor prognosis among various cancers, and early detection is critical for patient survival [43]. Pancreatic cancer usually presents as a hypovascular mass, but in the early stages it is often difficult to detect with diagnostic imaging, CT or MRI, due to its small size and poor contrast [44]. Virtual monochromatic images with 70 keV or less in photon-counting CT significantly improves conspicuity of pancreatic cancer in both, pancreatic and portal venous phase, compared to energy-integrating detector CT [45] (Fig. 8). In addition, pancreatic cancer is a highly invasive tumor [46], and it is important to determine the extent of its invasion on the images before treatment. The high spatial resolution of photon-counting CT may also be useful in evaluating tumor invasion [47].

#### Intraductal papillary mucinous neoplasm

**Fig. 9:**
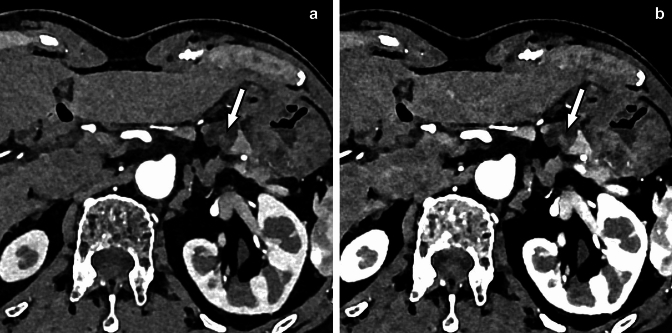
A cystic lesion with a mural nodule (arrows) is seen in the tail of the pancreas on the pancreatic parenchymal phase (arterial phase) photon-counting CT images at 70 (**A**) and 50 keV (**B**). The cystic lesion is consistent with an intraductal papillary mucinous neoplasm. The 50 keV image (**B**) shows the enhancement of the mural nodule more clearly than the 70 keV image (**A**). Such a nodule is a sign of malignancy *(i.e.*, high-risk stigmata)

Intraductal papillary mucinous neoplasm is the most common cystic neoplasm of the pancreas [48]. The increased attention to intraductal papillary mucinous neoplasm is due to its unique features of malignant progression [49]. Photon-counting CT can detect more pancreatic cysts than conventional energy-integrating detector CT [50]. The Fukuoka consensus proposes two-tiered criteria for predicting malignancy in intraductal papillary mucinous neoplasm, "high-risk stigmata" and "worrisome features" [51]. For patients with "high-risk stigmata" consisting of obstructive jaundice, enhancing mural nodules, and main duct dilatation ≥ 10 mm, immediate surgical resection is recommended [51]. Therefore, accurate evaluation of these findings on imaging is necessary. Imaging with photon-counting CT may be particularly helpful in the accurate evaluation of small structures such as enhancing mural nodules (Fig. 9).

### Vascular imaging

#### Vascular anatomy evaluation

**Fig. 10:**
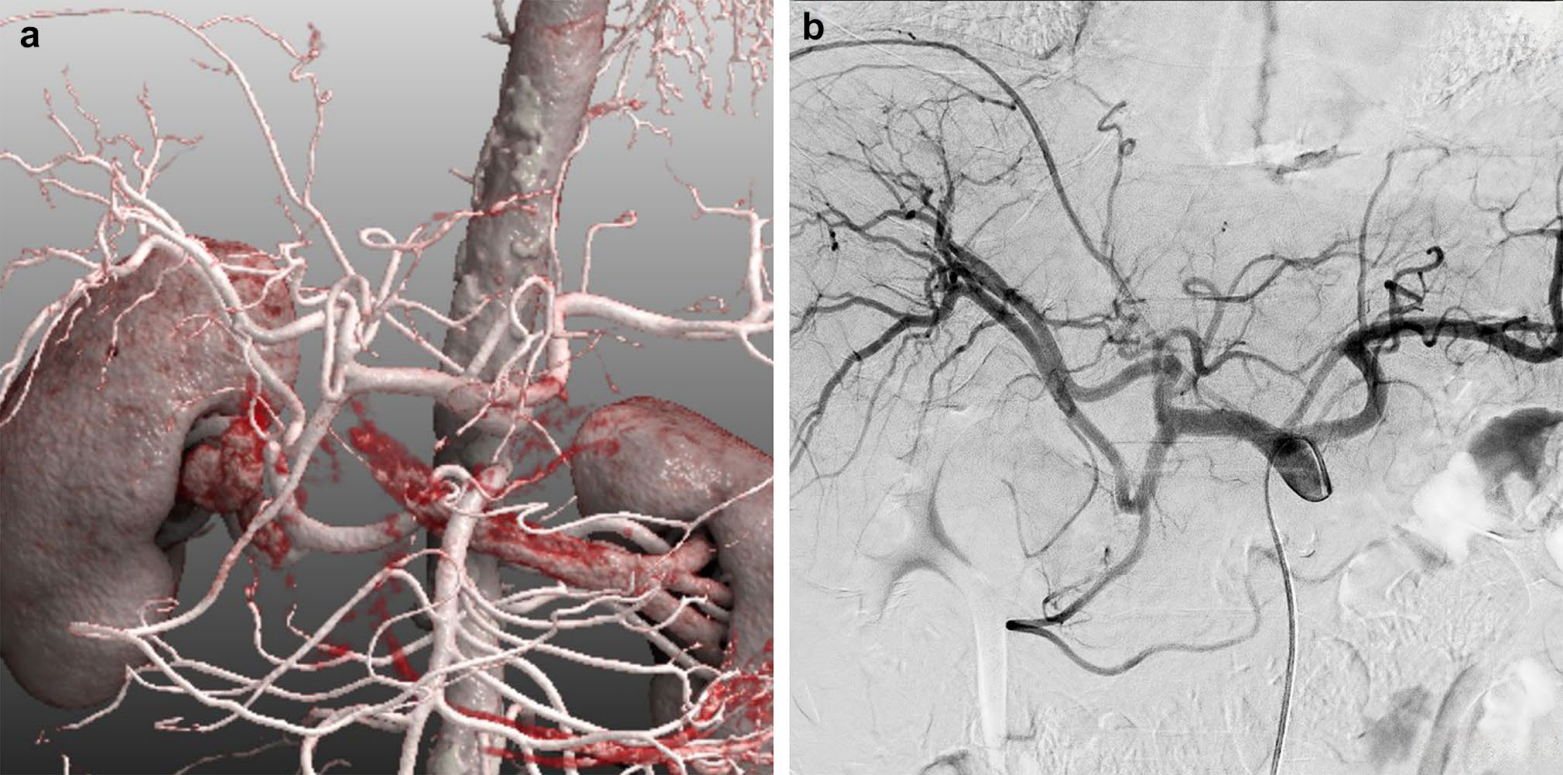
Photon-counting CT is also excellent for CT angiography (**A**) because of its superior spatial resolution. In this case, hepatocellular carcinoma was treated by transarterial chemoembolization, and celiac angiography was also performed during the procedure (**B**). CT angiography adequately delineated the hepatic artery to the periphery, which may facilitate selective catheter insertion into the hepatic artery branches

When performing laparoscopic surgery, the surgeon must have extensive preoperative information about the vascular anatomy to plan the procedure [52]. Visualization of vascular variants on preoperative imaging such as CT angiography can help identify these vessels and prevent fatal vascular injury [53]. Assessment of vascular anatomy by CT angiography is also useful in the preprocedural evaluation of arterial embolization procedures. Photon-counting CT shows considerable improvements in several aspects of vascular imaging [54] (Fig. 10). Dillinger et al. reported that virtual monochromatic images at 60–70 keV provide the best objective and subjective image quality in terms of vessel contrast [55].

#### Thrombosis

**Fig. 11:**
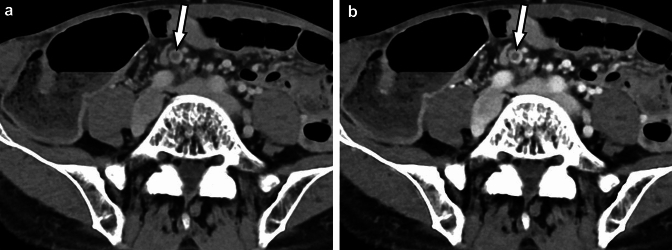
A small thrombus in the mesenteric vein (arrows) is visualized on the photon-counting CT images at 70 (A) and 50 keV (B). The 50 keV image (B) shows the thrombus with higher contrast than the 70 keV image (A). Photon-counting CT is excellent for visualization of venous thrombus

Contrast-enhanced CT is also useful in the diagnosis of thrombosis. With photon-counting CT, even small thrombi can be clearly visualized, contributing to an accurate diagnosis [56] (Fig. 11).

### Challenges

Investigation on the diagnosis of abdominal disease using photon-counting CT is still in its early stages and several challenges have been identified.

One of these may be the determination of optimal imaging parameters for the diagnosis of abdominal lesions. Since the type of image to be obtained depends on the purpose of the examination, it may be necessary to consider optimal settings for different clinical situations. The situations may be divided into pre-treatment examination, routine examination, and examination of patients with renal failure. Each of these situations requires improved diagnostic performance, lower radiation doses, and lower contrast doses, respectively. The large variety of parameters (e.g., radiation dose, reconstruction kernel, level of iterative reconstruction, energy level of VMI) also complicates the problem. These factors need to be properly organized to define reasonable imaging conditions.

Another issue may be the lack of evidence to show whether photon-counting CT significantly improves the ability to detect and diagnose disease, especially when compared to conventional CT systems. In such cases, due attention should be paid to whether the conventional CT being compared is spectral images similar to that of photon-counting CT or non-spectral images.

Further investigations will be needed to resolve these issues.

## Conclusion

Photon-counting CT provides a high contrast-to-noise ratio for abdominal CT imaging and may improve the ability to visualize small or low-contrast lesions. In addition, photon-counting CT may offer the opportunity of reducing radiation exposure dose.
